# Development of Antibody–Oligonucleotide Complexes for Targeting Exosomal MicroRNA

**DOI:** 10.3390/pharmaceutics12060545

**Published:** 2020-06-12

**Authors:** Asako Yamayoshi, Shota Oyama, Yusuke Kishimoto, Ryo Konishi, Tsuyoshi Yamamoto, Akio Kobori, Hiroshi Harada, Eishi Ashihara, Hiroshi Sugiyama, Akira Murakami

**Affiliations:** 1 Chemistry of Functional Molecules, Graduate School of Biomedical Sciences, Nagasaki University, 1-14 Bunkyo-machi, Nagasaki-shi, Nagasaki 852-8521, Japan; bb55620002@ms.nagasaki-u.ac.jp (S.O.); tsuyoshi.yamamoto@nagasaki-u.ac.jp (T.Y.); 2 PRESTO, Japan Science and Technology Agency (JST), 4-1-8 Honcho, Kawaguchi, Saitama 332-0012, Japan; 3 Faculty of Molecular Chemistry and Engineering, Kyoto Institute of Technology, Matsugasaki, Sakyo-ku, Kyoto 606-8585, Japan; y.kishimoto21@gmail.com (Y.K.); r.konishi0610@gmail.com (R.K.); akobori@kit.ac.jp (A.K.); am56365646@gmail.com (A.M.); 4 Department of Chemistry, Graduate School of Science, Kyoto University, Kitashirakawa-Oiwakecho, Sakyo-ku, Kyoto 606-8502, Japan; hs@kuchem.kyoto-u.ac.jp; 5 Laboratory of Cancer Cell Biology, Graduate School of Biostudies, Kyoto University, Yoshida-Konoecho, Sakyo-ku, Kyoto 606-8501, Japan; harada.hiroshi.5e@kyoto-u.ac.jp; 6 Department of Clinical and Translational Physiology, Kyoto Pharmaceutical University, Misasagi, Yamashina-ku, Kyoto 607-8414, Japan; ash@mb.kyoto-phu.ac.jp

**Keywords:** exosome, microRNA, nucleic acid drug, drug delivery system, antibody

## Abstract

MicroRNAs in exosomes (exosomal miRNAs) are considered as significant targets for cancer therapy. Anti-miR oligonucleotides are often used for the functional inhibition of miRNAs; however, there are no studies regarding the regulation of exosomal miRNA functions. In this study, we attempted to develop a novel drug delivery system using anti-exosome antibody–anti-miR oligonucleotide complexes (ExomiR-Tracker) to hijack exosomes to carry anti-miR oligonucleotides inside exosome-recipient cells. We found that ExomiR-Tracker bound to the exosomes, and then the complexes were introduced into the recipient cells. We also found that anti-miR oligonucleotides introduced into the recipient cells can exhibit inhibitory effects on exosomal miRNA functions in vitro and in vivo. We believe that our strategy would be a promising one for targeting exosomal miRNAs.

## 1. Introduction

MicroRNAs (miRNAs) are a type of non-coding RNA that induce the post-transcriptional gene silencing of their target genes and regulate a wide range of biological processes, including apoptosis, differentiation, metabolism, and cell proliferation [1,2]. Recent studies have reported that the aberrant expression of miRNAs is associated with many pathological disease processes [3,4,5]. Furthermore, miRNAs have been identified in exosomes [6]. Exosomes are nano-sized vesicles (50–100 nm in diameter) released by a variety of cell types. Exosomal RNAs can be taken up by neighboring or distant recipient cells [7]. It has been reported that such miRNAs (exosomal miRNAs) regulate gene expression in the recipient cells [5,8,9,10]. Therefore, exosomal miRNAs can be considered as significant targets for cancer therapy [8,9,10].

**Figure 1:**
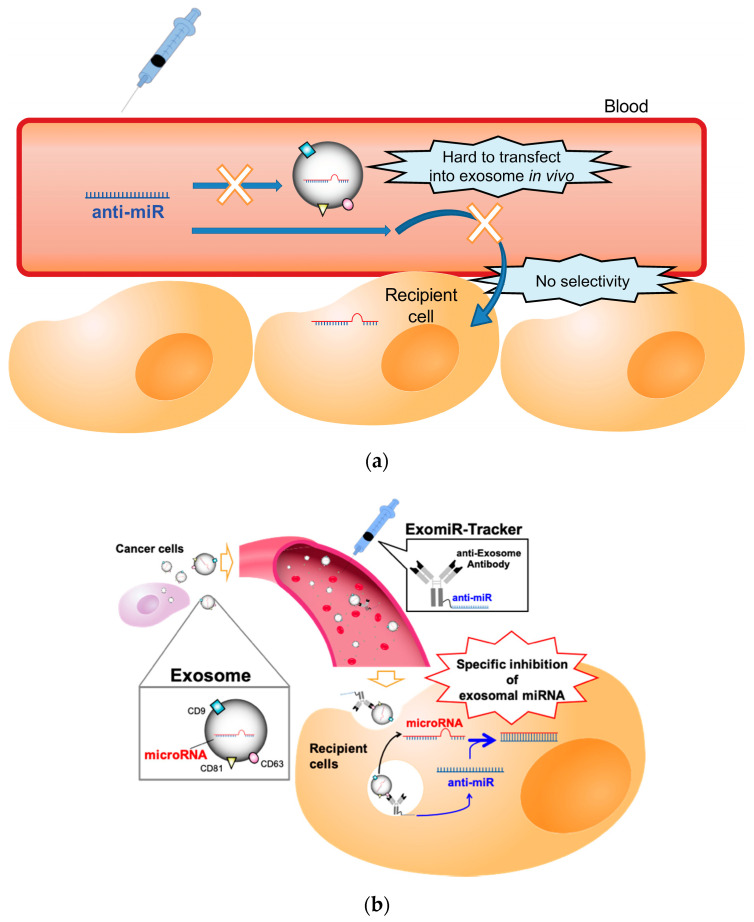
Schematic representation of the concept of this study: (**a**) The problems for targeting exosomal miRNA with anti-miR oligonucleotides in vivo. (**b**) ExomiR-Tracker binds onto the surface of the exosome, leading to incorporation into the cells and the subsequent inhibition of exosomal miRNA functions.

In general, anti-miR oligonucleotides, which have a sequence complementary to miRNAs, are often used for the functional inhibition of miRNAs [11,12,13]; however, there is no useful strategy to regulate the function of miRNAs in exosomes in vivo, because exosomal miRNAs are encased in an exosome, and exosomes do not have any uptake mechanisms for oligonucleotides, such as a cellular endocytosis pathway [14]. Therefore, it is hard to introduce anti-miR oligonucleotides into the exosome in vivo (Figure 1a). Additionally, recent studies have revealed that exosomes are not incorporated into all cell types [15]. For example, Hoshino and co-workers reported that integrin clusters on an exosome determine the organotropic uptake of the exosomes [16]. If we want to introduce therapeutic nucleic acids (TNAs) into exosome-recipient cells before the exosome enters the cells in vivo, we have to identify the in vivo distribution profiles of the target exosome and have to develop functional oligonucleotides for specific delivery to all the kinds of recipient cells targeted by the exosome.

This study attempted to develop a novel drug delivery system using anti-exosome antibody–oligonucleotide complexes (ExomiR-Tracker) that enable the functional inhibition of exosomal miRNAs (Figure 1b). In this system, ExomiR-Tracker binds to the surface of an exosome and hijacks exosomes to carry anti-miR oligonucleotides inside the recipient cells. We then hypothesized that the ExomiR-Tracker–exosome complex can be introduced into the targeted cells, after which the anti-miR oligonucleotides of ExomiR-Tracker inhibit the function of the exosomal miRNAs in the recipient cells.

## 2. Materials and Methods

### 2.1. Preparation of ExomiR-Tracker

The cationized antibodies were obtained according to Ma’s report [17]. Briefly, the anti-CD63 antibody (Cosmobio, Tokyo, Japan) was thiolated using Traut’s reagent (Thermo Scientific, Waltham, MA, USA) in phosphate buffer (pH 8.0) containing 2 mM EDTA and then conjugated with Cys(Npys)-(D-Arg)_9_ (AnaSpec, Fremont, CA, USA). The anti-CD63 IgG-9r was purified by size-exclusion chromatography (Figure S2) and concentrated using Amicon-Ultra 40K (Merck Millipore, Burlington, MA, USA). The stoichiometry of thiol modification on the antibody was calculated based on the signals resulting from reaction with Ellman’s reagent (Thermo Scientific) (Figure S3). The introduction number of arginine moieties to IgG was estimated to be 2.8 molecules.

The anti-CD63 IgG-9r/anti-miR complex was obtained by mixing anti-CD63 IgG-9r and anti-miR in PBS at the indicated molar ratios and incubating the mixtures at room temperature for 20 min. The sequence of anti-miR21 [18] was as follows: 5′- U ^m^C A A ^m^C A U ^m^C A G T C U G A U A A G ^m^C U A -3′. The control sequence [18] was as follows: 5′- U T C U ^m^C C G A A C G U G T C A ^m^C G U T A U -3′ (plain: 2′-*O*-methly RNA, underlined: locked-nucleic acid (LNA)). The chemical modification patterns of those oligonucleotides are different from those of antagomirs [10].

### 2.2. Cell Lines

The oral squamous cell carcinoma cell line Cal27 (ATCC, Manassas, VA, USA) was maintained in Dulbecco’s Modified Eagle’s medium (DMEM) (FUJIFILM Wako, Osaka, Japan) supplemented with 10% heat-inactivated FBS in a 5% CO_2_ atmosphere.

### 2.3. Confocal Microscopy

For the evaluation of the cellular localization of anti-exosome IgG, cells were grown on a glass-bottom culture dish (Matsunami, Osaka, Japan) at 37 °C for 24 h and then were supplemented with Alexa647-labeled anti-exosome IgGs (Cosmobio). After a further 24 h incubation, the cells were washed with PBS and fixed with 4% paraformaldehyde. The dishes were mounted after staining the nuclei with Hoechst33342 (FUJIFILM Wako) and the actin with act-stain (Cosmobio); then, the cells were analyzed by confocal microscopy (Zeiss Upright LSM510: Carl Zeiss, Oberkochen, Deutch).

For the exosome-dependent cellular uptake of anti-CD63 IgG, the cells were grown as described above for 24 h. On the following day, the growth medium was removed and replaced with serum-free DMEM (Advanced-DMEM, Thermo Scientific), and then the cells were supplemented with Alexa647-labeled anti-CD63 IgG with or without Cal27 exosomes (50 mg/mL). After 12 h of incubation, the cells were fixed and stained according to the above procedures.

For ExomiR-Tracer, the cells were grown on glass-bottom culture dishes for 24 h, and then the cells were treated with ExomiR-Tracker containing TAMRA-labeled anti-miR ([antimiR] = 200 nM). After 24 h of incubation, the cells were fixed and stained in the same way as above.

### 2.4. Luciferase Reporter Assay

Cells were cultured in DMEM at 37 °C in 5% CO_2_. The cells were plated into 96-well plates (32 mm^2^/well) at a density of 4.5 × 10^4^ cells/mL in an antibiotic-free medium and cultured for 24 h. On the following day, the cells were treated with ExomiR-Trackers ([anti-miR] = 300 nM) for 24 h. After the incubation, the cells were transfected with pmirGLO (0.1 µg/well) or pmiR-21 reporter (0.1 µg/well) plasmids using Lipofectamine 2000 (Thermo Scientific) according to the manufacturer’s protocol. After 24 h of incubation, the cells were lysed and the luminescence activity of the lysate was measured with a dual luciferase assay kit (Promega, Madison, WI, USA).

### 2.5. Exosome Isolation and qRT-PCR

At 70% confluency in 100 mm culture dishes, cells were washed with PBS, and then the growth medium was replaced with Advanced-DMEM (Thermo Scientific). The cells were cultured under 20% O_2_ (normoxic) or 0.1% O_2_ (hypoxic) conditions, balanced with N_2_ in a three-gas incubator. After 48 h, the culture supernatant was collected and centrifuged at 300 × *g* for 10 min to remove cellular debris. Exosome isolation using the classical ultracentrifugation method was done according to a previous report [19].

Total RNA was isolated using a total exosome RNA isolation kit (Thermo Fisher Scientific) as per the manufacturer’s instructions and quantified using a Nanodrop-1000 (Thermo Fisher Scientific). In total, 40 ng of RNA was used for cDNA synthesis using a high-capacity RNA-to-cDNA synthesis kit (Thermo Fisher Scientific), where specific reverse transcription (RT) primers were used for U6 and miR-21, while random RT primers were used for cDNA synthesis for β-actin and GAPDH. Then, 5 μL of cDNA was used as a template for polymerase chain reaction (PCR) without dilution using a CFX96 touch real-time PCR detection system (Bio-Rad, Hercules, CA, USA) in a total 20 μL reaction volume that included 10 μL of SYBR green qPCR master mix (2×) containing specific forward and reverse primers sets. The thermal cycling conditions were as follows: cycle 1 at 95 °C for 10 min, and cycle 2 (× 40) at 95 °C for 10 s and 56 °C/60 °C for 45 s followed by melting curve detection. The detection of the fluorescence signal was represented in the form of the cycle threshold (Ct).

### 2.6. Scratch Assay

A scratch assay was performed to measure cell migration in vitro according to the Kroh’s report [20]. Briefly, cells were seeded onto fibronectin-coated 24-well dishes to create a confluent monolayer for 24 h. The cell monolayer was scraped in a straight line to create a scratch with a p200 pipette tip and then incubated with tumor-derived exosomes (20 mg/mL) and ExomiR-Trackers ([anti-miR] = 300 nM). The first image of the scratch was acquired, and the cells were cultured in the incubator at 37 °C for 24 h prior to the acquisition of the second image. The percentage of wound closure (%) was the migrated cell surface area/total surface area times 100.

### 2.7. In Vivo Study

Nude mice (females, 6 weeks of age) were obtained from Japan SLC Inc (Shizuoka, Japan). Cells were co-injected with ExomiR-Tracker ([anti-miR] = 300 nM) subcutaneously (5 × 10^6^ cells/100 uL PBS/mouse) into the back of nude mice (*n* = 6). The tumor sizes were monitored weekly by measuring the diameters using vernier calipers and calculated as πls^2^/6, where l is the long side and s is the short side.

## 3. Results and Discussion

### 3.1. Cellular Uptake of Anti-Exosome Antibodies

**Figure 2:**
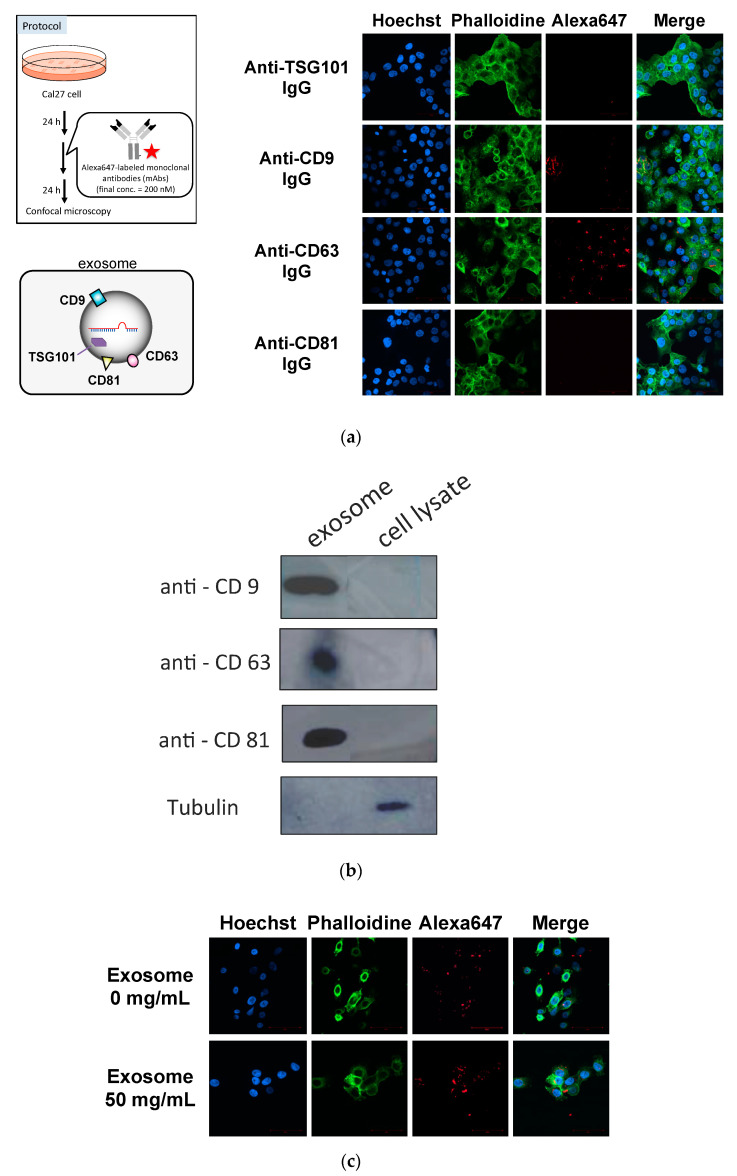
Cellular localization of fluorescently labeled anti-exosome antibodies (after 24 h of incubation) (**a**), analysis of the expression levels of antigens on the surfaces of exosomes and whole cell lysates by Western blotting (**b**), and exosome-dependent cellular uptake of anti-CD63 IgG in serum-free medium (after 12 h of incubation) (**c**).

First, we determined whether the anti-exosome antibody could be introduced into the recipient cells. As antigens of anti-exosome antibody, CD9, CD63 and CD81, which are known as surface markers of exosomes, were selected [20]. Anti-TSG101 antibody was selected as the control IgG because TSG101 is located inside of the exosomes [20]. Alexa647-labeled antibodies were added to the medium and incubated for 24 h. Then, the cells were fixed and analyzed using confocal microscopy (Figure 2a). It was found that the anti-CD63 antibody was successfully incorporated into cells, whereas the fluorescent signals were low for the anti-CD9 and anti-CD81 antibodies. Similar results were obtained in the case of HeLa cells (Figure S1).

We also evaluated the expression levels of CD9, CD63 and CD81 in exosomes (Figure 2b) and found that the expression levels of each protein were almost the same (slightly low in the case of CD63). On the other hand, the amounts of CD9, CD63 and CD81 in whole cell lysates were not at detectable levels. Furthermore, to assess whether the cellular uptake of anti-CD63 IgG was exosome-dependent, anti-CD63 IgG was incubated with cells with or without exosomes in serum-free medium. After 12 h of incubation, the cellular uptake of anti-CD63 IgG was observed (Figure 2). The fluorescent signals of Cal27 cells incubated with both anti-CD63 IgG and exosomes were much stronger than those in the case without exosomes (about four-folds higher). These results suggest that the anti-CD63 antibody interacted with CD63 antigen on the surface exosome and was delivered to the recipient cells. Based on these results, we selected the anti-CD63 antibody as a component of ExomiR-Tracker.

### 3.2. Cellular Uptake and Localization of ExomiR-Trackers

**Figure 3:**
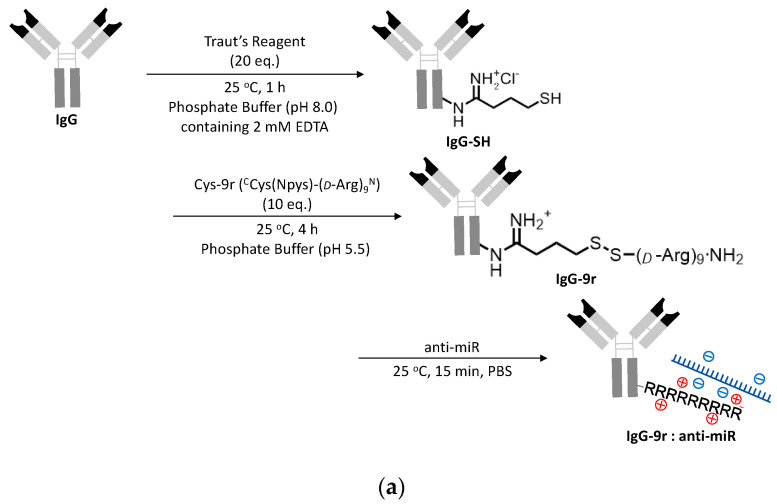

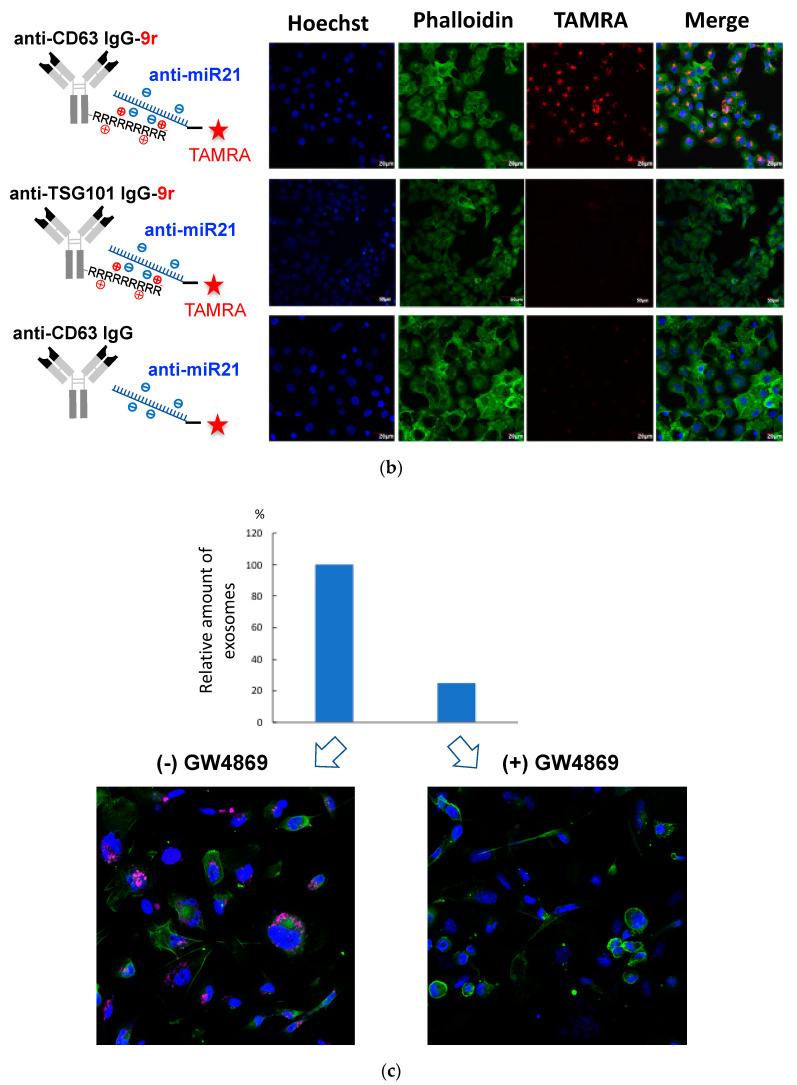
Successful incorporation of ExomiR-Tracker into the recipient cells: (**a**) The synthetic scheme of ExomiR-Tracker. (**b**) Cellular localization of ExomiR-Trackers with TAMRA-labeled anti-miRs. (**c**) Number of exosomes produced by Cal27 cells after GW4869-treatment (above) and the cellular uptake of ExomiR-Trackers with TAMRA-labeled anti-miR oligonucleotides.

The molecular design and the synthesis of ExomiR-Trackers are shown in Figure 3a. We selected a 9-mer of D-arginine to enhance the endosomal escape of the anti-miR oligonucleotides [17]. First, an amino residue of IgG was reacted with Traut’s Reagent (2-iminothiolane HCl), yielding thiolated IgG (IgG-SH). Next, IgG-SH was incubated with Cys(Npys)-(D-Arg)_9_ peptide (Cys-9r) to form each other via disulfide bonds (IgG-9r). The introduction number of arginine to IgG was estimated to be 2.8 molecules from the results in Figure S3 (the details are described in the supporting information). Finally, IgG-9r was incubated with anti-miR oligonucleotides. The functional assessments of this construct were confirmed by an electrophoretic mobility shift assay (Figure S4).

We confirmed the cellular uptake and localization of ExomiR-Tracker by confocal microscopy (Figure 3b). For the assessment, a TAMRA-labeled fully chemical-modified anti-miR was used to exhibit a high nuclease resistance and high binding affinity to the target miR-21. In the case of the [anti-CD63-9r/anti-miR] complex, fluorescence signals were successfully observed within Cal27 cells. By contrast, in the case of [anti-TSG101-9r/anti-miR] and non-cationized anti-CD63 antibody, no fluorescent signals were observed. To elucidate the mechanism, GW4869 was used to inhibit exosome generation (Figure 3c). Upon treatment with GW4869, the number of exosomes released from Cal27 cells was drastically decreased (Figure 3c, above), and the fluorescent signals of ExomiR-Trackers were also decreased by treatment with GW4869 (Figure 3c below), suggesting that ExomiR-Tracker is incorporated into the recipient cells by binding onto the surfaces of exosomes.

### 3.3. Evaluation of Inhibitory Effects of ExomiR-Trackers on MiR-21 Functions

**Figure 4:**
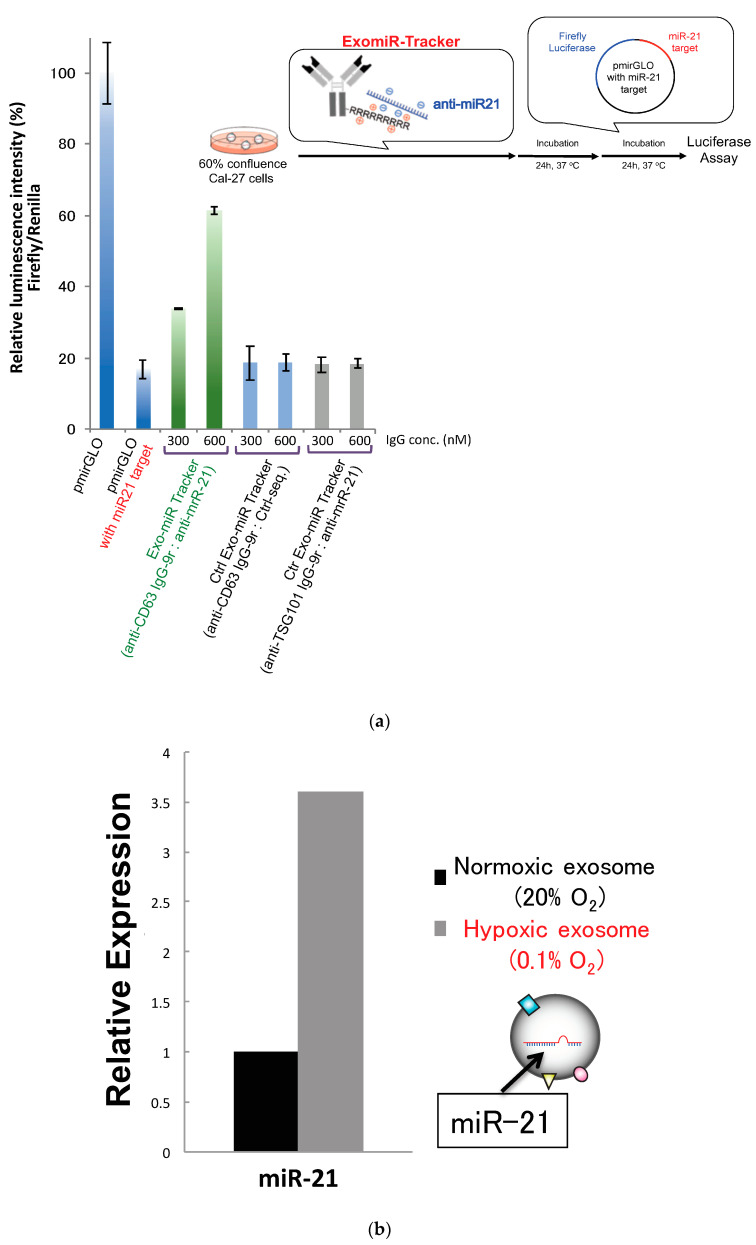

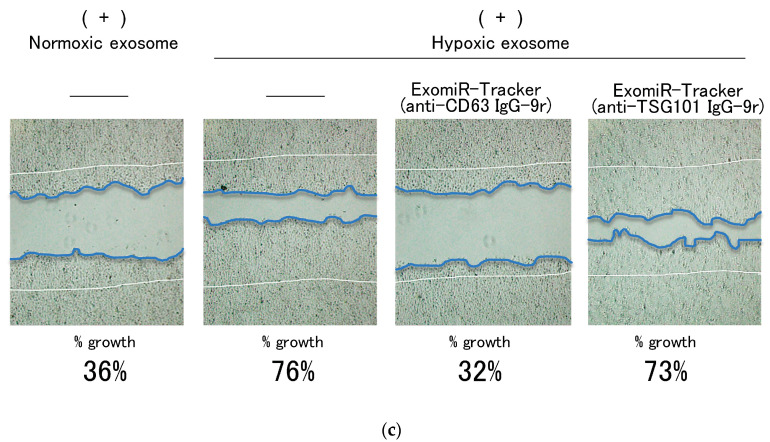
Successful inhibition of exosomal miRNA by ExomiR-Tracker in the recipient cells: (**a**) Evaluation of the inhibitory effects by ExomiR-Trackers on miR-21 by a luciferase assay. (**b**) Amount of exosomal miR21 under the hypoxic and normoxic conditions. (**c**) ExomiR-Tracker successfully inhibited exosomal miR-21 function in Cal27 cells.

We then moved on to evaluating the inhibitory effects of the ExomiR-Tracker against miRNA functions according to a previous report (Figure 4a) [18]. As the target of anti-miR oligonucleotides, miR-21, which is known as one of most famous onco-miRNAs, was selected [10]. We prepared fully chemical-modified anti-mi21 oligonucleotides as described above [18]. The inhibitory effects of ExomiR-Trackers against miR-21′s functions are shown in Figure 4a. In this system, the luminescence intensity of firefly luciferase is already downregulated by endosomal miR-21. If an anti-miR inhibits the RISC function, it is expected that the luminescence intensity would be recovered. We found that the luminescence intensity of ExomiR-Tracker-treated cells was recovered. By contrast, in the control cases (Ctrl ExomiR-Trackers), no recovery effects were observed. These results suggest that ExomiR-Tracker successfully inhibits the function of miR-21 in Cal27 cells. Under hypoxic conditions, the amount of exosomal miR-21 is upregulated, and cell growth and migration are promoted [10]. We confirmed the amount of exosomal miR-21 by real-time RT-PCR (Figure 4b), and the data indicated that exosomal miR-21 was upregulated 3.5-fold under hypoxic conditions compared with that under normoxic conditions.

We further evaluated the inhibitory effects of ExomiR-Tracker on the cellular growth driven by exosomal miR-21(Figure 4c). The scratch assay indicated that a hypoxic exosome, but not a normoxic exosome, could promote cell migration and proliferation. ExomiR-Tracker successfully inhibited the cell growth to 32%—almost equal to that in the normoxic conditions. By contrast, ExomiR-Tracker constructed with the anti-TSG101 antibody did not have an inhibitory effect. These results indicate that ExomiR-Tracker inhibits the cancer cell growth promoted by exosomal miRNA.

**Figure 5:**
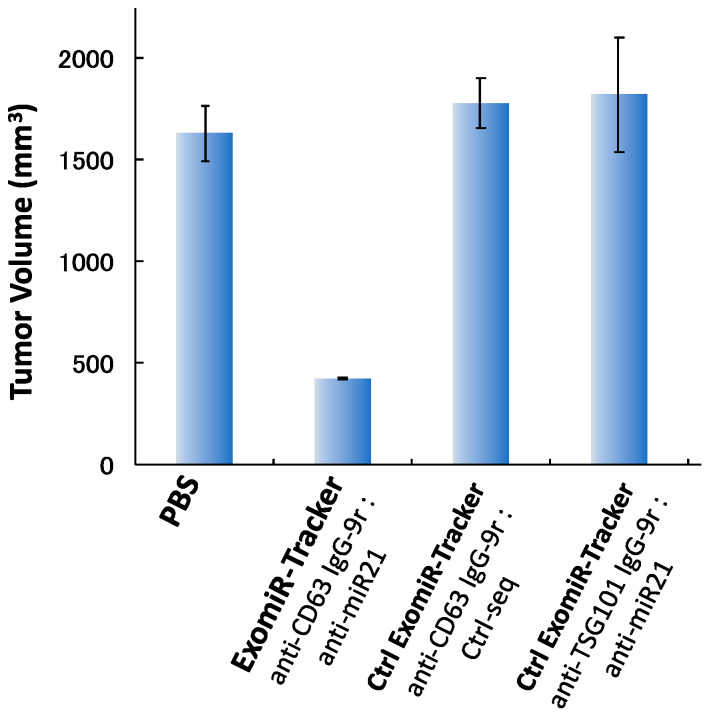
ExomiR-Tracker inhibited exosomal miR-21 function in vivo. Anti-tumorigenesis effects of ExomiR-Trackers in nude mice.

Finally, we examined the anti-tumorigenic effect of ExomiR-Tracker in vivo. Cal27 cells and ExomiR-Trackers were subcutaneously co-injected into the hind foot, and after 4 weeks, tumor volumes were measured. In the control cases, [anti-CD63 IgG-9r/Ctrl-seq] and [anti-TSG101 IgG-9r/anti-miR21], no inhibitory effects were observed compared to the PBS groups. By contrast, we found that ExomiR-Tracker successfully inhibited tumorigenesis in vivo (Figure 5). From these results, it is suggested that ExomiR-Tracker can inhibit tumorigenesis in a sequence-specific manner in vivo.

## 4. Conclusions

We successfully demonstrated that ExomiR-Tracker can be incorporated into recipient cells and inhibits the function of exosomal miR-21 to prohibit cancer cell growth in vitro and in vivo. Our results suggest that this strategy allows us to use exosomes as natural cargo for TNAs without exosome isolation. Exosomes are considered as potential cargo for the delivery of TNAs, and there are several methods for the introduction of TNAs into isolated exosomes from plasma or cell cultures; however, despite the potential significance of exosomes, they continue to be challenging cargo mainly due to the lack of efficient exosome isolation technology and TNA-loading methods [14]. The anti-miR21 used in this study is a TNA, and it would be expected that it should exhibit specific inhibitory effects on gene expression. It must be noted that the leaking of anti-miR-21 might be induce some side effects. In the near future, we plan to develop a new ExomiR-Tracker which has anti-miR oligonucleotides via covalent bonding to antibodies and will examine its effects in vivo. We have already found that an Alexa647-labeled anti-CD63 antibody was selectively accumulated in tumors following intravenous injection (Figure S5). We believe that our strategy would be a promising one for delivering various kinds of nucleic acid drugs to cancer cells.

## Supplementary Materials

The following are available online at https://www.mdpi.com/1999-4923/12/6/545/s1. Figure S1: Cellular localization of fluorescently labeled anti-exosome antibodies in HeLa cells (after 24 h of incubation). Figure S2: SEC analysis of IgG-9r. Figure S3: Standard SH (acetyl Cysteine) calibration curve using Ellman’s Reagent (a), and quantification of the introduction number of SH groups to IgG (b). Figure S4: Gel mobility shift assay of the [anti-CD63 IgG-9r/anti-miR] complex (a). Figure S5: In vivo distribution of Alexa-647 labeled anti-CD63 antibody.
