# Supplementary material for: Development of Antibody–Oligonucleotide Complexes for Targeting Exosomal MicroRNA
Source: Pharmaceutics. 2020 Jun 12;12(6):545. doi: 10.3390/pharmaceutics12060545 (PMC7355672; doi:10.3390/pharmaceutics12060545)
Supplement: Supplementary file 1 [file pharmaceutics-12-00545-s001.pdf]

pharmaceutics

Supplementary Materials: Development of Antibody–Oligonucleotide Complexes for Targeting Exosomal MicroRNA

Asako Yamayoshi, Shota Oyama, Yusuke Kishimoto, Ryo Konishi, Tsuyoshi Yamamoto, Akio Kobori, Hiroshi Harada, Eishi Ashihara, Hiroshi Sugiyama and Akira Murakami

Table of Contents

1. Cellar uptake of anti-exosome IgGs in HeLa cells (Figure S1)
2. Preparation of ExomiR-Tracker (Figure S2, Figure S3)
3. Gel Shift Assay (Figure S4)
4. *In vivo* distribution of anti-CD63 antibody (Figure S5)

Results and Discussion

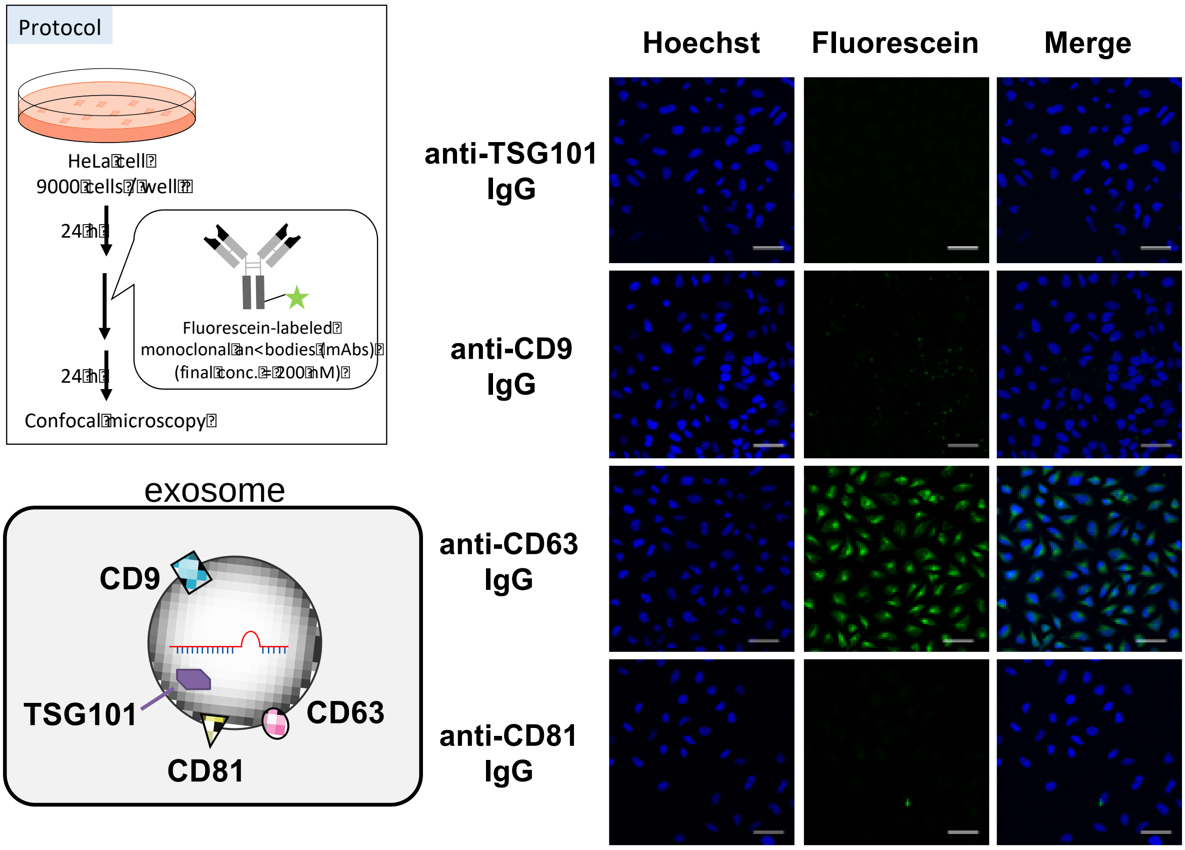

Figure S1. Cellular localization of fluorescent-labeled anti-exosome antibodies in HeLa cells (after 24 h incubation) .
[Fluorescent labeled IgG] = 200 nM

（a）**IgG alone**

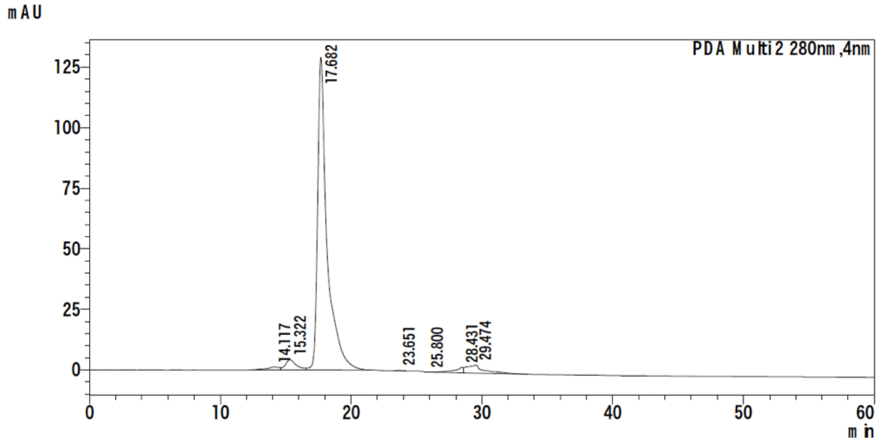

（b）**Cys(Npys)-(D-Arg)$_9$ alone**

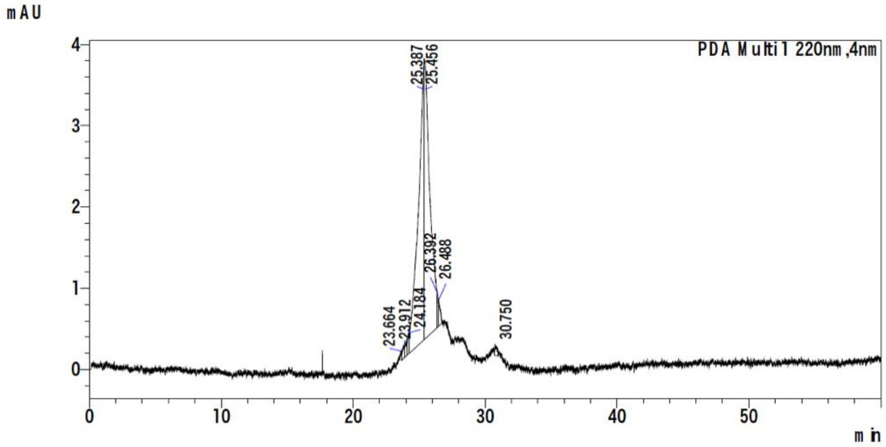

（c）**IgG-9r**

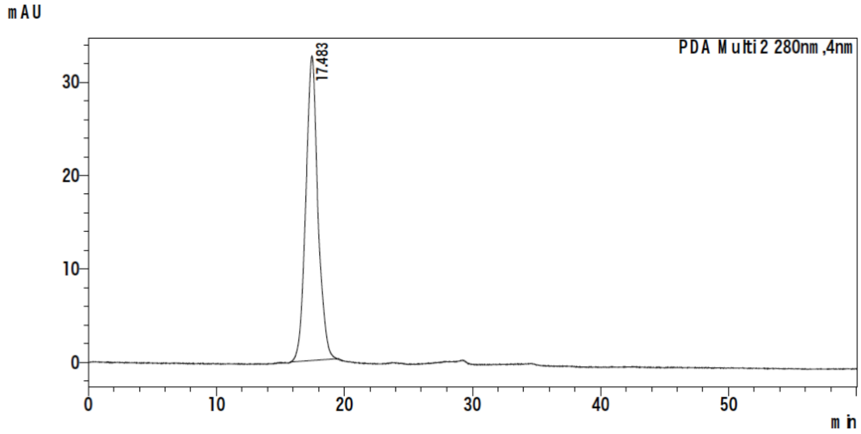

Figure S2. SEC analysis of IgG-9r
Column: TSKgel G3000SWXL (TOSOH, Japan), 0.5 mL/min, PBS, r.t..

Quantification of introduction number of D-(Arg)$_9$ peptides to IgG

The stoichiometry of thiol modification on the IgG was calculated based on signals resulting from reaction with Ellman's reagent (Thermo Scientific). Ellman's reagent (5,5'-dithio-bis-[2-nitrobenzoic acid]) is used to estimate sulfhydryl groups in a sample by comparing to a standard curve of a sulfhydryl-containing compound such as cysteine.

Figure S3 shows standard SH (acetyl cysteine) calibration curve using Ellman's reagent (a) and quantification of introduction number of SH groups to IgG (b). First, we conducted quantification of SH groups of IgG-SH (Figure 3a) and confirmed that introduction number of SH to IgG -SH was 3.4 groups. Next, after the conjugation of D-(Arg)9, we evaluated the remained number of SH of IgG-9r and confirmed that the number is 0.6 groups. Furthermore, from these results in Figure S3, we estimated the introduction number of arginine to IgG were 2.8 molecules.

(a)

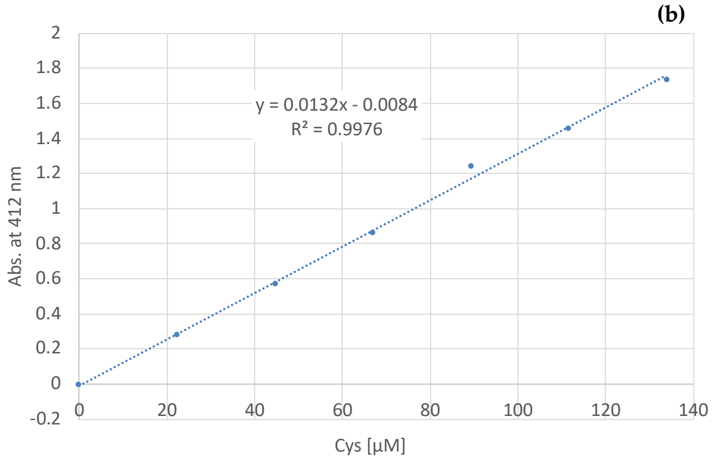

(b)

	5 eq.	10 eq.	20 eq.
Abs. at 412 nm	0.290	0.349	0.630
TNB* conc. (μM)	20.7	24.9	45.0
IgG conc. (μM)	15.0	10.8	13.5
free SH/IgG (①)	1.4	2.3	3.4

*TNB: 5-Thio-2-nitrobenzoate

after conjugation of D-(Arg)$_9$

	5 eq.	10 eq.	20 eq.
Abs. at 412 nm	0.260	0.193	0.134
TNB conc. (μM)	18.6	13.8	9.60
IgG conc. (μM)	18.0	12.8	16.0
free SH/IgG(②)	1.0	1.1	0.6
Arg/IgG(①−②)	0.4	1.2	2.8

Figure S3. Standard SH (acetyl Cysteine) calibration curve using Ellman's Reagent (a), and quantification of introduction number of D-(Arg)$_9$ peptides to IgG (b)

Gel Shift Assay

The anti-CD63 IgG-9r : anti-miR complex was obtained by mixing anti-CD63 IgG-9r and anti-miR in PBS at the indicated molar ratios and incubating the mixtures at RT for 20 min. The binding of TAMRA-labeled anti-miR to anti-CD63 IgG-9r was analyzed on 6% Native-PAGE in 0.5X TBE (Figure S4).

(a)

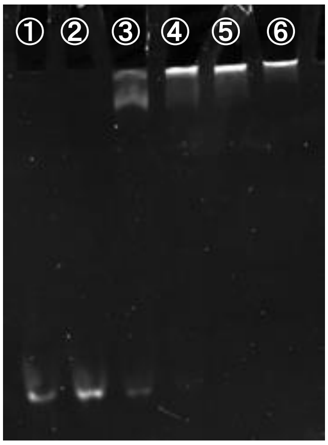

Lane	Sample (molar ratio)
1	anti-miR alone
2	anti-miR + IgG (1:1)
3	anti-miR + IgG-9r (1:1)
4	anti-miR + IgG-9r (1:2)
5	anti-miR + IgG-9r (1:4)
6	anti-miR + IgG-9r (1:8)

anti-miR = 5 pmol

(b)

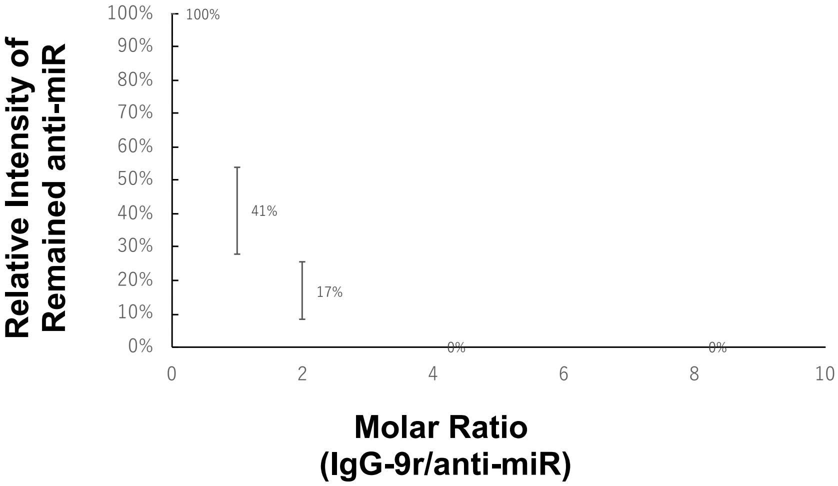

Figure S4. Gel mobility shift assay of [anti-CD63 IgG-9r : anti-miR] complex (a) and quantification of relative intensity of remained anti-miR (b)

***In vivo* distribution**

The Nude mice (females, 6 weeks of age) were obtained from Japan SLC Inc. Cal 27 cells were injected subcutaneously (5 X 10^6 cells/100 uL PBS/mouse) into the back of nude mice (n = 6). After the 4 weeks later, Alexa-647-labeled anti-CD63 antibody was injected intravenously (3uM /100 uL PBS/mouse). The mice were dissected and measured fluorescence intensity at 647 nm by using IVIS Spectrum In Vivo Imaging System (PerkinElmer).

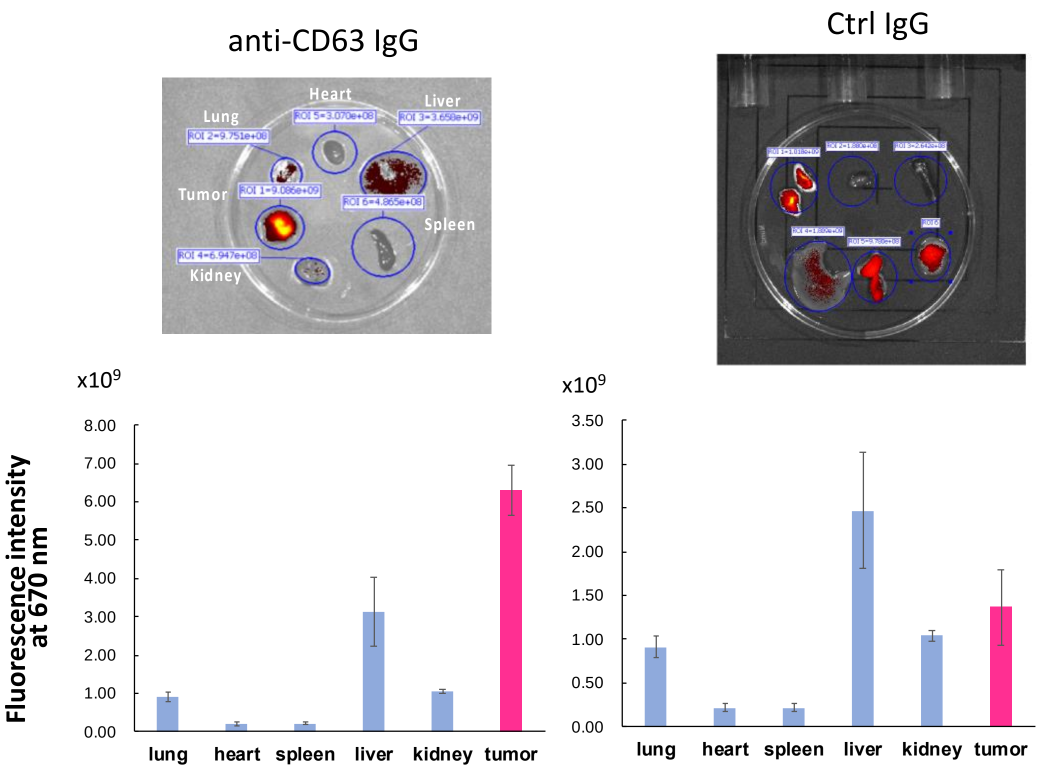

Figure S5. *In vivo* distribution of Alexa-647 labeled anti-CD63 antibody.
